# Cardiac dynamics: a simplified model for action potential propagation

**DOI:** 10.1186/1742-4682-9-50

**Published:** 2012-11-29

**Authors:** Angelina Peñaranda, Inma R Cantalapiedra, Jean Bragard, Blas Echebarria

**Affiliations:** 1 Departament de Física Aplicada, Universitat Politècnica de Catalunya. BarcelonaTech, Av. Dr. Marañon 44-50, 08028 Barcelona, Spain; 2 Departamento de Física y Matemática Aplicada, Universidad de Navarra, Irunlarrea s/n, 31080 Pamplona, Spain

## Abstract

This paper analyzes a new semiphysiological ionic model, used recently to study reexitations and reentry in cardiac tissue [I.R. Cantalapiedra *et al*, PRE **82** 011907 (2010)]. The aim of the model is to reproduce action potencial morphologies and restitution curves obtained, either from experimental data, or from more complex electrophysiological models. The model divides all ion currents into four groups according to their function, thus resulting into fast-slow and inward-outward currents. We show that this simplified model is flexible enough as to accurately capture the electrical properties of cardiac myocytes, having the advantage of being less computational demanding than detailed electrophysiological models. Under some conditions, it has been shown to be amenable to mathematical analysis. The model reproduces the action potential (AP) change with stimulation rate observed both experimentally and in realistic models of healthy human and guinea pig myocytes (TNNP and LRd models, respectively). When simulated in a cable it also gives the right dependence of the conduction velocity (CV) with stimulation rate. Besides reproducing correctly these restitution properties, it also gives a good fit for the morphology of the AP, including the notch typical of phase 1. Finally, we perform simulations in a realistic geometric model of the rabbit’s ventricles, finding a good qualitative agreement in AP propagation and the ECG. Thus, this simplified model represents an alternative to more complex models when studying instabilities in wave propagation.

## Introduction

Cardiac action potentials (APs) are electrical signals that trigger the synchronous contraction of the heart. As such, the regular propagation of the action potential is necessary for ensuring a correct heart functioning. APs are produced as a result of ion currents that cross the cell membrane, producing a net depolarization or repolarization of the membrane as different currents are invoked in response to the transmembrane voltage changes. The currents are produced by the movement of individual ions through ion channels, which are specialized pore-forming proteins that span the cell membrane, forming a pathway for ions to cross it. Each type of channel is highly selective for a specific type of ion. The most common intracellular ion concentrations considered in cardiac models are calcium, sodium, and potassium. Channelopaties due to mutations that modify the ion channel function can perturb the form of the action potential, sometimes leading to cardiac dysfunctions or altered AP propagation.

Following the pioneering mathematical description of the action potential by Hodgkin and Huxley [1] in neuronal cells, the first cardiac models considered, in much the same way, the changes in the transmembrane potential produced by a sum of ionic gated currents. As more refined experimental data of the different currents and their dynamics became available, more complex models of the AP for specific cardiac cells have been proposed [2]. The quest of new models has been especially noticeable for several animal species, as rabbit [3], guinea pig [4], rat [5], or dog [6]; and, most recently, for humans [7-10]. Nowadays, one can find very detailed models in the literature where the description of ionic channel gating is given in terms of Markov processes [11], which permit to link specific mutations to the model parameters. A known drawback of the Markov formulation is the increasing complexity of the models and consequently the very high computational cost associated to the simulation of few beats in a realistic heart geometry. Clearly, a highly realistic ionic model is ideal as a test bench, allowing to safely test multiple hypotheses and making predictions without incurring any cardiac risk for the patient, or to check conditions not easily reproducible in experiments. But, despite their more realistic description, these models are often difficult to analyze, let alone amenable for deep mathematical analysis.

In order to tackle the problem of modeling AP propagation in the computer, simplified models have also been proposed, from the very simple but unphysiological [12-14], to the so-called semiphysiological models [15-19], somewhere in between the former and the extremely detailed physiological models mentioned earlier. These semi-physiological models preserve particular sets of properties of the action potential, as for instance, the dependence of the AP duration (APD, time during which the voltage is above a certain threshold, characterizing the duration of the excited state) or the conduction velocity (CV, propagation speed of the AP pulse) on the stimulation period, known as restitution curves. In this respect, these can be viewed as mesoscopic models, that bridge the gap between dynamics at the molecular level (ion channel gating) and whole heart description, being still manageable for both computation and theoretical analysis.

A widely used semiphysiological model is the one by Fenton and Karma [15], a three-variable model of the cardiac action potential. This model uses three transmembrane currents, i.e., fast inward, slow inward, and slow outward, which resemble the set of physiological sodium, calcium, and potassium currents, respectively. In the Fenton-Karma model, the variables are the transmembrane potential and two gating variables that are used to regulate the inactivation of the fast inward and slow inward currents, respectively. Despite its simplicity, the model can reproduce fairly the action potential duration (APD) and conduction velocity (CV) restitution curves of more complex models or experiments. However, it fails to reproduce accurately the AP morphology. This is a strong drawback of the Fenton-Karma model. In fact, the model was originally created in order to describe the propagation and properties of scroll waves. The underlying idea is that wave behavior is determined by the restitution properties of the system. Thus, it has also been useful in the study of cardiac alternans (a beat to beat modification in the duration of the AP, at fast pacing rates), where the onset and subsequent evolution of alternans has been related to the shape of the restitution curves. However, more recent results have shown that models with the same restitution properties, but different AP morphologies may give different onsets for alternans [17]. The reason is that the dynamics of alternans is also affected by electrotonic currents, that depend crucially on the form of the action potential [20]. Furthermore, electrotonic currents are also a determinant factor for the occurrence of re-excitation and phase 2 re-entry in cases where the duration of the action potential is non-homogeneous in the heart tissue [21]. Typical examples are Brugada [22], or long QT [23] syndromes, that may be caused by fast or delayed repolarization, respectively.

With the aim of providing models that fit better the AP morphology, a modification of the three-variable Fenton-Karma model has been proposed in [19]. This new model includes an additional gate that modulates the slow inward current, in a fashion that resembles the effect of the fast outward potassium current (*I*_*to*_) in physiological models. This allows to reproduce the notch in phase 1 of the AP typical of epicardial cells. In contrast to [19], in the present paper we specifically include the effect of the *I*_*to*_. Following the same idea as in the Fenton-Karma model, we divide the currents not by their carrier ion, but by their function: inward or outward currents, and among these, slow and fast currents, to a total of four. With this assumption, we show that it is possible to reproduce all the important characteristics of AP propagation and morphology. Here, we specifically compare the results with more realistic models of electrical activity in human myocytes [8] and guinea pig heart ventricles [4], and with available experimental data [24], including action potential shape for different pacing rates, and different types of myocytes, i.e. epicardial; endocardial; and midmyocardial cells.

The present model has been recently used to study reexcitations in tissue presenting large dispersion of repolarization [25]. There, it was shown that reexcitation is due to a slow pulse, induced by the L-type calcium current, that propagates from the region of long APDs to the region of short APDs until it encounters newly excitable tissue. An interesting advantage of studying this mechanism using this simplified model was the possibility to obtain the characteristics of this slow pulse analytically.

The paper is organized as follows: in the next section we introduce the equations of the model. Then, the AP morphology is studied, obtaining the proper parameters matching with experiments and two detailed models: Luo-Rudy (LRd) model for guinea-pig [4], and ten Tusscher *et al.* (TNNP) model of human ventricular cells [8]. A discussion of the different currents and gates follows, as well as a more detailed comparison with other semiphysiological models. In the next section, we perform simulations of AP propagation in a three-dimensional model of the ventricles. In the concluding section, we provide some future lines of research opened by this paper.

## Mathematical formulation of the simplified model

The cellular transmembrane potential $\tilde{V}$ (physical units are mV) satisfies the following equation:

(1) $$\frac{\partial \tilde{V}}{\partial t}=\nabla \cdot (D\nabla \tilde{V})-\frac{I_{\text{ion}}-I_{\text{stim}}}{C_{m}}\;\;,$$

where ∇ is the standard nabla operator, $D=\sigma /(\tilde{S}C_{m})$ is the anisotropic diffusion tensor (units: cm^2 ^ms^−1^), *σ* is the electrical conductivity (units: mS cm^−1^), $\tilde{S}\equiv S/\text{Vol}$ is the cell surface to volume ratio (units: cm^−1^) and *C*_*m*_ is the membrane capacitance per unit area (≈1 *μ*F cm^−2^). The simulations in a one-dimensional cable are carried out by replacing the spatial derivative term $\nabla \cdot (D\nabla \tilde{V})$ by its one-dimensional counterpart $D\partial_{\text{xx}}^{2}\tilde{V}$ in Equation (1). In the three-dimensional simulations, one must take into account the inhomogeneous and anisotropic behavior of the diffusion parameter [26]. All computer simulations have been performed using a simple Euler forward algorithm (for time integration) and finite spatial discretization in order to ensure current conservation up to double precision arithmetic. The time step is fixed to *dt *= 0*.*01 ms. The uniform spatial discretization mesh used in the simulations was set to *dx *= 0*.*02 cm for 1D and 2D, and *dx *= 0*.*025 cm for 3D simulations. The current *I*_*ion *_is the sum of the ionic currents flowing across the cell membrane including the ones exchanged by the pumps through active transport (units are *μ*A cm^−2^) and *I*_*stim*_ is an applied external stimulation current. To stimulate the cell we apply it during 1 ms with an amplitude of 1.5 times the threshold stimulation value.

Realistic models include ion currents corresponding to all the existing ion channels experimentally found in the cell membrane. In the present simplified model, we decompose the total membrane current into only four components, i.e., *I*_*ion *_=* I*_*fi*_ + *I*_*si*_ + *I*_*so*_ + *I*_*to*_, where the sum contains a fast inward current *I*_*fi*_(Na^+^ current plus the fast part of the Ca^2 + ^ current); a slow inward Ca^2+ ^current, *I*_*si*_; a slow outward time-independent K^+ ^current, *I*_*so*_; and a fast transient outward K^+ ^current, *I*_*to*_. The model does not consider the intra- and extracellular concentrations of different ions, and therefore the effect of the pumps is implicitly included in the previous currents. At this point, let us remind that in the Fenton-Karma model, the three variables of the model are the membrane potential *V*(*xt*), the inactivation gate *h*(*t*) of the fast inward current, and the inactivation gate *f*(*t*) of the slow inward current; steady-state activation is assumed for both of these currents. In the present paper, we supplement the Fenton-Karma model variables with two additional dynamical variables associated with the activation and inactivation of the *I*_*to *_current. The latter current being the essential novelty with respect to the three variable model of Fenton and Karma [15]. Let us insist that it is crucial to take into consideration this fast transient outward current to obtain accurately the characteristic notch in the phase 1 of the AP, most noticeable in epicardial myocytes. This current was experimentally studied in human hearts by Nabauer *et al*. [27] and Li *et al*. [24] and has been suggested to contribute significantly to regional electrophysiological heterogeneity in myocardial cells and tissue of several animal species. In particular, it is well known that the human ventricle shows substantial transmural heterogeneity in AP morphology related to transient outward properties. Furthermore, this current is sometimes related to the occurrence of phase-2 reentry [28,29].

In the rest of the paper, in order to alleviate the mathematical notation, it is convenient to rewrite Eq. (1) in dimensionless form by defining the membrane dimensionless potential $V=(\tilde{V}-{\tilde{V}}_{\text{res}})/\Delta \tilde{V}$, which varies roughly between zero and one and where ${\tilde{V}}_{\text{res}}≃-85$ mV is the resting potential of the polarized membrane and $\Delta \tilde{V}=100$ mV. One also defines the scaled currents $J_{\text{fi}}=I_{\text{fi}}/[C_{m}\Delta \tilde{V}]$ (and similar expressions for the others currents: *J*_*so*_; *J*_*to*_ ; *J*_*si*_, which have all dimension of inverse time and units are ms^−1^). The explicit expressions for these currents are given by:

(2) $$\;J_{\text{fi}}=-g_{\text{fi}}\;h\;m_{\infty }(V_{\text{fi}}-V)\;,$$

(3) $$\;J_{\text{si}}=-g_{\text{si}}\;d_{\infty }\;f\;f_{\infty }^{\prime },$$

(4) $$J_{\text{to}}=g_{\text{to}}\;r\;s(V-V_{\text{to}}),$$

(5) $$J_{\text{so}}=g_{\text{so}}\;k_{\infty },$$

where *V*_*fi *_and *V*_*to *_are parameters indicating the reversal potentials of sodium and potassium respectively. The four gate variables obey the classical saturation kinetics:

(6) $$\dot{h}=[h_{\infty }(V)-h]/\tau_{h}(V)\;,\;\;\dot{f}=[f_{\infty }(V)-f]/\tau_{f}(V),$$

(7) $$\dot{r}=[r_{\infty }(V)-r]/\tau_{r}(V)\;,\;\;\dot{s}=[s_{\infty }(V)-s]/\tau_{s}(V),$$

where upper dots denote differentiation with respect to time. The expressions for the steady-state values of the gates appearing in the current formulations, and the time constants of exponential functions with which they converge, are:

$$\begin{array}{ll} \;m_{\infty } & =(V-V_{c})\;\Theta (V-V_{c})\;,h_{\infty }=f_{\infty }=s_{\infty }=1-\Theta (V-V_{c})\;,\\ \;d_{\infty } & =\Theta (V-V_{c})\;(1+\text{tanh}[\beta_{1}(V-V_{1})])/2\;,\\ \;f_{\infty }^{\prime } & =(1-\text{tanh}[\beta_{2}(V-V_{2})])/2\;,\\ \;k_{\infty } & =V/V_{c}+\Theta (V-V_{c})(1-V/V_{c})\;,\\ \;r_{\infty } & =\Theta (V-V_{r})\;,\\ \;\tau_{h} & =\tau_{h_{+}}-(\tau_{h_{+}}-\tau_{h_{-}})\Theta (V-V_{c}),\\ \;\tau_{f} & =\tau_{f_{+}}-(\tau_{f_{+}}-\tau_{f_{-}})\Theta (V-V_{c}),\\ \;\tau_{r} & =\tau_{r_{-}}+(\tau_{r_{+}}-\tau_{r_{-}})\Theta (V-V_{r}),\\ \;\tau_{s} & =\tau_{s_{+}}-(\tau_{s_{+}}-\tau_{s_{-}})\Theta (V-V_{c}), \end{array}$$

where Θ(*.*) is the Heaviside function, *V*_*r *_= 0*.*6, *V*_*to *_= 0 and the rest of the parameters of our model are given in Table 1 for human experimental epi, endo, midmyocardium, and TNNP and LRd numerical models. Note that in the above expressions a + subscript refers to the time associated for a given gate to converge to one and, conversely, a − subscript refers to the time associated for a given gate to converge to zero. The determination of the numerical values of the parameters will be explained in the next section.

**Table 1:** Values of the parameters for the present model

**Parameters**	**Epi **[24]	**Endo **[24]	**M-cell **[24]	**TNNP **[8]	**LRd **[4]	**Units**
$\tau_{h_{+}}$	17.9	10.8	11.3	90.5	25.8	ms
$\tau_{h_{-}}$	11.4	10.8	1.88	6.61	0.950	ms
$\tau_{f_{+}}$	123	355	101	43.1	488	ms
$\tau_{f_{-}}$	183	52.3	228	181	25.4	ms
$\tau_{r_{+}}$	2.51	7.54	2.15	13.5	5.10	ms
$\tau_{r_{-}}$	2.00	6.07	0.371	2.20	13.1	ms
$\tau_{s_{+}}$	57.0	29.1	4.67	99.9	300	ms
$\tau_{s_{-}}$	10.6	10.4	1.75	4.34	7.11	ms
*g*_*fi*_	4.00	1.72	2.62	14.0	10.0	ms^−1^
*V*_*fi*_	1.46	1.24	1.60	1.18	1.20	–
*g*_*so*_	0.0161	0.00891	0.0278	0.0498	0.0316	ms^−1^
*g*_*si*_	0.176	0.414	0.103	0.138	2.32	ms^−1^
*β*_1_	3.99	22.8	27.1	11.8	6.90	–
*β*_2_	1.56	2.95	6.12	8.05	6.36	–
*V*_1_	0.529	0.522	0.668	0.200	0.453	–
*V*_2_	0.386	0.596	1.08	1.02	0.828	–
*g*_*to*_	2.10	0.300	1.36	9.82	2.50	ms^−1^
*V*_*c*_	0.130	0.130	0.130	0.350	0.380	–
*V*_*s*_	0.3	0.6	0.3	0.6	0.6	–

## Results

### Comparison with experimental results and detailed electrophysiological models

One of the main goals of the present model is to reproduce the AP morphology measured in experiments, or obtained with more complex mathematical models, for different types of myocytes and different pacing rates. Hence, the present model is an extension of the Fenton-Karma model, but with the ability of reproducing the AP morphology and, particularly, the notch in phase 1 faithfully.

We have validated our model in two ways: firstly, we have fitted experimental APs for human endo-, mid-, and epicardial ventricular cells choosing the appropriate model parameters. Secondly, we have also fitted the AP morphologies of two of the most currently used theoretical models in the literature: ten Tusscher *et al*. [8] for human ventricular myocytes (TNNP), and Luo-Rudy [4,30] for guinea pig (LRd), but also widely used in other contexts. We have fitted the parameters of our model such that the AP morphologies, as well as the correct conduction velocities, at different pacing rates were obtained. The model parameters are determined by using a standard nonlinear optimization routine from Matlab based on the Levenberg- Marquardt algorithm, that minimizes the square of the difference between the model action potential and the objective template. One subtlety of the optimization process used here is that we have imposed a slightly larger weight in the zone corresponding to the notch of the AP that is mostly influenced by the *J*_*to *_current. The codes used for the fit, as well as more information about the model can be found in [31].

#### Comparison with experimental human cell ventricular morphologies

**Figure 1:**
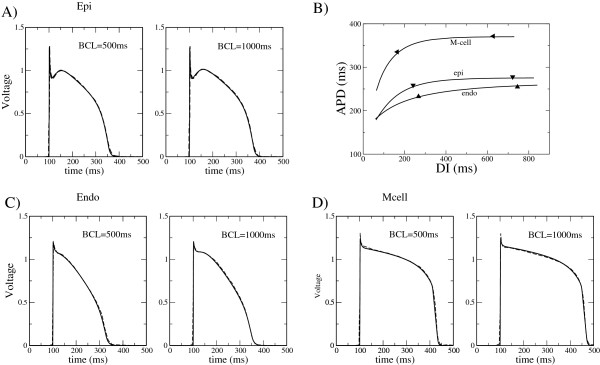
**AP morphologies calculated from the simplified model and compared to the experimental data by Li et al. **[24]** for different myocytes: A) epicardium, C) endocardium, D) mid-myocardium.** Dashed lines represent the experimental data at two pacing rates, i.e., BCL=500 and 1000 ms. The APs obtained from the simplified model are represented by solid lines. **B**) Restitution curve APD(DI) for the parameters corresponding to different fits. The values by Li *et al*. are indicated by filled triangles.

As a first use of our model, we show in Figure 1 the comparison between experimental data of AP morphologies and the present model. We have fitted the model to the experiments of Li et al. [24], that were done at pacing rates of 1 and 2 Hz. As it is clear from Figure 1, the model reproduces well the deep notch observed in epicardial cells; the more accentuated action potential duration of M-cell; as well as the variation of the action potential duration with the stimulation rate in all cases. Indeed, the inclusion of the transient outward current in the model allows to fit perfectly the notch in the transmembrane voltage after the initial depolarization phase.

Let us mention that human data provided by other authors [24,27,32,33] differ slightly. The cause of the discrepancies being presumably due to the variation in the temperature of experiment, the heart rate stimulation, and if the right/left heart side ventricle were used. In the present paper, we considered the experiments done by Li et al. [24] because in the same paper the authors present the three different types of myocytes at several physiological heart rates.

**Figure 2:**
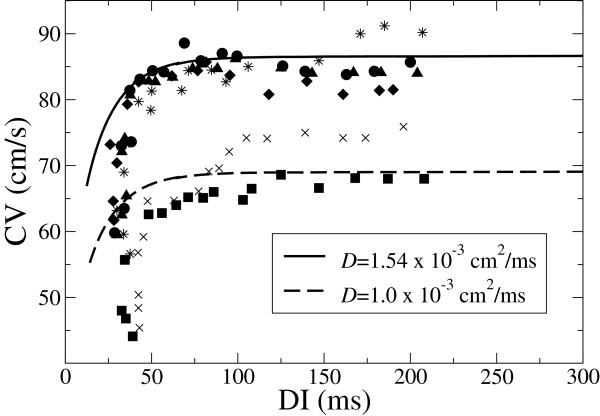
**CV-restitution curve for the parameter set corresponding to epicardial cells and diffusion constants*D *= 1*.*54 × 10**^**−3**^**cm**^**2**^**/ms (as in Tusscher *et al***[8]**) and*D *= 1 × 10**^**−3**^**cm**^**2**^**/ms **[15]**.** For comparison we also show the results by Yue et al, for various experimental set-ups [34].

In Figure 2 we compare typical experimental conduction velocities for epicardial cells [34] and the ones obtained with our model, for two different values of the diffusion coefficient. Although the dispersion in the experimental data is significant (maybe because of different diffusivities or slightly different sodium conductance), the results provided by our model agree well with typical observed conduction velocities in epicardial cells.

#### Comparison with detailed ventricular electrophysiological models

**Figure 3:**
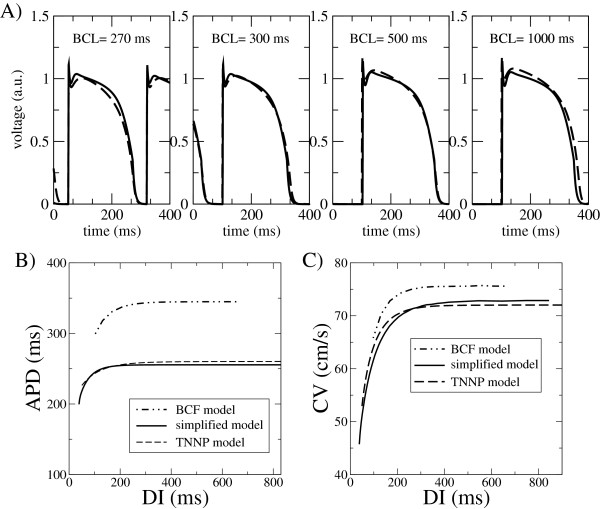
**Comparison of different models. A)** APs morphologies from the simplified model (solid line) and those obtained from the simulation of the TNNP model (long dashed line). Four different pacing rates (BCL=270, 300, 500, 1000 ms) have been used for determining the parameter set of our model. Restitution curves **B)** APD(DI) and **C)** CV(DI) for our simplified model and the TNNP model. For completeness we also include the results of the 5 variable BCF model, with their parameter values for the fit to the TNNP model.

Following the same protocol, we have also fitted our model to the epicardial human ventricular model developed by ten Tusscher *et al*. [8]. In this case, since we have access to data of both APD and CV-restitution, we perform a simultaneous optimization search of the model parameters for both curves, obtained simulating Eq. (1) in a 1D cable. Here the comparison of the AP morphology has been performed at four pacing periods, i.e., 270, 300, 500, 1000 ms. Due to the memory of the TNNP model the data of the AP comparison are obtained when the stationary state is achieved. We also include the CV at those periods in the objective function that we minimize. This is an optimization problem in a highly dimensional parameter space, with presumably many local minima. It is therefore difficult to ascertain if a given solution of the minimization procedure is the best possible one. However, as it was the case with the experimental data, the comparison between the simplified model and the TNNP model is very good, as it can be seen in Figure 3. For comparison sake we also show the results obtained with the model proposed by Bueno *et al*. [19] (BCF model), with the parameter set they provide for their fit to the TNNP model. The large disagreement between the BCF and TNNP models suggests that there is most presumably a typo error in some of the BCF parameters [19].

Another important physiological model is the LRd model for guinea pig [4,30] (where we consider the last version of the model, obtained from [35]). In Table 1 we provide the parameter values for the fit to the LRd model. In this case, the comparison between our model and the LRd model was again done at four following pacing periods: 100, 250, 500 and 1000 ms. The LRd dynamic model (using *D *= 10^−3^ cm^2^/ms) gives an approximate propagation speed of 52 cm/s at large DI. This value compares well with the actual velocity ∼50 cm/s obtained with our model with parameter values fitted to the LRd model (see Table 1).

### Gates dynamics and transmembrane currents

To obtain a better understanding of the newly proposed model, we will briefly discuss the shape of the different currents entering into the formulation of our model. The currents in our simplified model have all dimension of inverse of time (units ms^−1^). In order to perform a direct comparison with the currents in more complex models they must be multiplied by the factor $C_{m}\Delta \tilde{V}$ to obtain the corresponding dimensional current in *μ*A cm^−2^. 

**Figure 4:**
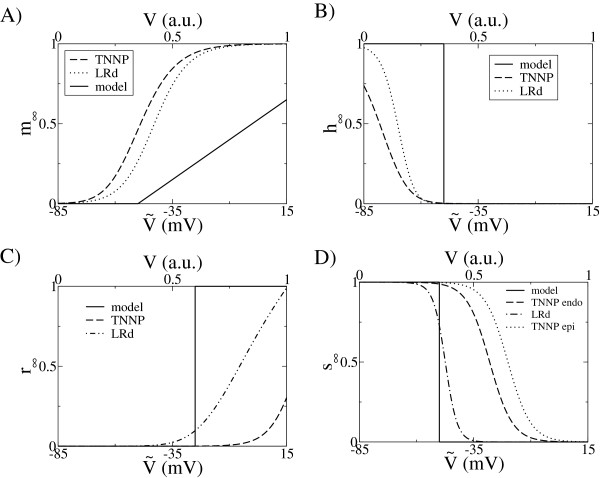
**Comparisons of gates dynamics.** Top panels: fast inward current: *m* gate (left) corresponds to the activation gate; *h* gate (right) corresponds to the fast inactivation gate. In both sides, the simplification of the present model relative to more complex models is clearly seen. Our model is represented by a solid line, the TNNP model is indicated by a dashed line and the LRd model is indicated by a dotted line. Lower panels: Comparison of the steady state values of the activation and inactivation gates of the transient outward current *I*_*to*_.

• *J*_*fi*_: *fast inward current*. It corresponds mainly to the Na^+ ^current in more detailed electrophysiological models. For instance, in the TNNP model [8], it is expressed by

(8) $$I_{\text{Na}}/C_{m}=G_{\text{Na}}m^{3}\text{hj}(\tilde{V}-E_{\text{Na}}),$$

where *m* is an activation gate, *h* is a fast inactivation gate, and *j* is a slow inactivation gate. The sodium conductance in the TNNP model is equal to *G*_*Na *_= 14*.*838 nS/pF. Straightforward dimensional analysis allows to compare this value for the conductance to the one in our model *g*_*fi *_= 14 ms^−1^(nS/pF ≡ ms^−1^, see the value given in the ninth row and fourth column of Table 1). For the gates dynamics, some simplifications are done in the model. The dynamics of the activation gate *m* is fast (in realistic models *τ*_*m *_∼ 0*.*1 ms), so we take its steady state value, given in Figure 4 (although this may lead to a decrease in the numerical accuracy of the model, see [36]). Therefore, it opens at *V *>* V*_*c*_, (for the fit to TNNP, we set *V*_*c *_= 0*.*35, equivalent to $\tilde{V}=-50$ mV). The product of the steady state of the inactivation gates *h* and *j* is modeled by a step function Θ(*V*−*V*_*c*_). The time constants for opening and closing are $\tau_{h_{+}}=90.5$ ms and $\tau_{h_{-}}=6.61$ ms, respectively, which again are of the order of magnitude of those found in the TNNP model. The Nernst potential *E*_*Na*_ is typically in the range *E*_*Na *_≃ [40−70] mV, while in our case we have set *V*_*fi *_= 1*.*18, corresponding to $\tilde{V}=33$ mV. The time constant $\tau_{h_{+}}$ is related to the recovery of the gates from inactivation, and gives the CV restitution properties of the propagating AP.

• *J*_*si*_: *slow inward current*. This corresponds approximately to the Ca^2+ ^current in more detailed electrophysiological models, responsible for the formation of the plateau (phase 2) in the AP. The principal component is the current *I*_*CaL*_, that, in the TNNP model is given by

(9) $$I_{\text{CaL}}/C_{m}=G_{\text{CaL}}\;\;d\;f\;f_{\text{Ca}}\frac{4\tilde{V}F^{2}}{\text{RT}}\frac{Ca_{i}e^{2\tilde{V}F/\text{RT}}-0.341Ca_{0}}{e^{2\tilde{V}F/\text{RT}}-1}$$

This current depends on the intra- and extracellular Ca^2+^ concentrations denoted by *Ca*_*i *_and *Ca*_0_, respectively. The current defined by Equation (9) possesses three gates, two voltage dependent gates *d* and *f*, and a third one *f*_*Ca *_that depends on the calcium concentration. In the present model we do not include the calcium concentration dynamics, and therefore all the gates are only voltage dependent. We assume that voltage activated gate *d* is fast, therefore we neglect its dynamics and rather take its steady-state value. Formally, it means that the dynamics of calcium current is slaved to voltage dynamics, so the last part of *I*_*CaL *_gives an effective voltage activated gate. In the model, the dependence on voltage is given by two sigmoidal functions that determine the voltage range at which this current is active. In conclusion, all the dynamics associated with calcium lies in the voltage inactivation gate *f*, that operates at a time scale of $\tau_{f_{+}}∼43$ ms for opening, and $\tau_{f_{-}}∼181$ ms, for closing. The time constant $\tau_{f_{-}}$ is related with the AP duration, while $\tau_{f_{+}}$ determines the AP restitution properties.

• *J*_*to*_: *transient outward current*. This current corresponds to the transient K^+ ^current responsible for the notch in the action potential. In the TNNP model it is expressed by :

(10) $$I_{\text{to}}/C_{m}=G_{\text{to}}\text{rs}(\tilde{V}-E_{k}).$$

For the reversal potential we take *V*_*to *_= 0, which corresponds to $\tilde{V}=-85$ mV, while typical values in the literature are *E*_*K *_≃ −100 mV. For the gates, once again we adopt the simplified formulation through step functions for opening (or closing) at *V*_*r *_= 0*.*6, corresponding to a potential $\tilde{V}=-25$ mV, similar to the TNNP model. In our model, the dynamics of the gate *s* is similar to the inactivation gate of Na^+^, and closes in a time scale of ∼4 ms. For the activating gate *r*, we have a time scale for opening of ∼13 ms. A comparison of the steady state values of the gates as a function of the transmembrane potential is shown in Figure 4.

• *J*_*so*_: *slow outward current*. This current corresponds to the slow (mainly K^+^) repolarization currents. Among the slow potassium currents, one can distinguish: *I*_*Ks*_, *I*_*Kr*_, *I*_*K*1_,.. but all in all, their sum is almost constant. We therefore maintain the assumption of the FK model that this current depends on a single gate at steady state.

**Figure 5:**
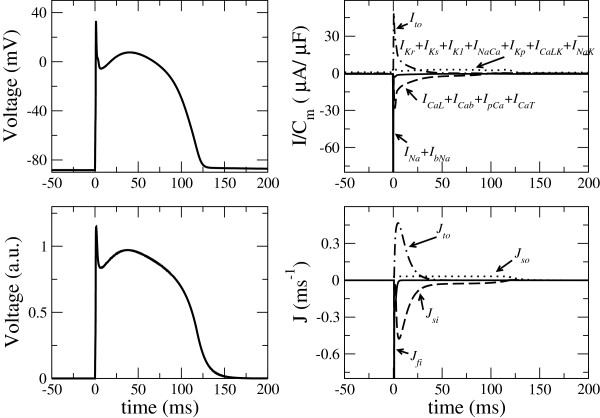
**Comparison of the currents between the LRd model (top) and our simplified model (bottom), for the corresponding parameters given in Table **1** (fifth column).**

In Figure 5 we show the AP and currents for the LRd model. We have grouped the currents into four groups corresponding to the classification in our model. It should be noted that our fast inward current *J*_*fi*_ corresponds to the sum of the sodium current *I*_*Na*_ plus the fast part of the calcium current *I*_*CaL*_, in more realistic electrophysiological models, as for example in the LRd. The current corresponding to the pumps *I*_*NaCa *_and *I*_*NaK *_has been grouped with the sum of the potassium currents, that collectively correspond to the *J*_*so*_ of the simplified model. We should stress that these currents do not have a direct electrophysiological meaning, since we are dealing with a reduced model, but a comparison with the currents in realistic models helps to clarify the meaning of the different currents in the simplified model.

## Comparison with other simplified models

In this section we compare our model with other existing simplified models proposed in the literature. Two of the simplest models of cardiac dynamics contain only two variables. These are the models by Mitchell and Schaeffer [16] and by Aliev and Panfilov [14]. In both cases the equation for the transmembrane voltage includes just two currents, one outward *J*_*out*_ and one inward *J*_*in*_, such that *dV*/*dt *=* J*_*in*_−*J*_*out*_. Besides the equation for the transmembrane voltage, there is an additional equation for a gate variable that, in [16] modulates the inward current, while in [14] modulates the outward current. Despite their extreme simplicity, these models present restitution properties and morphologies that resemble those of cardiac cells (except for the missing notch in phase 1 of the AP). Interestingly enough to mention, in the model by Mitchell and Schaeffer it is even possible to calculate the APD restitution curve analytically, under certain constraints in the parameters. This, together with the conduction velocity at a given pacing rate (or the maximum CV) fixes univocally all the coefficients in the model. Once this is done, there is no more freedom to fit the shape of the CV-restitution curve. Unfortunately, for typical restitution curves found in human cardiac cell, the previous constraints are not fulfilled and, thus, the simple expressions for the APD-restitution do not hold.

The Mitchell-Schaeffer two variable model is a simplification of the Fenton-Karma model [15]. In the latter the inward current is split into a fast, *J*_*fi*_ and a slow *J*_*si *_component. This model is able to fit both APD and CV-restitution curves at the same time, however it does not fit properly the morphology. A further simplified model was proposed by Bueno *et al*[19], including an additional gate that modulates the slow inward current, mimicking the effect of the transient outward current *I*_*to*_. This modification implies the need to introduce different values of the parameters at different levels of the transmembrane voltage, through the inclusion of Heaviside functions. In the present paper, we follow a similar approach but, instead of modulating the slow inward current as it is done in the BCF model, we explicitly introduce a fast outward current similar to the physiological *I*_*to*_, an approach that we reckon more natural. This approach, for instance, has been useful to study Brugada syndrome [25,29], where the loss of the dome is achieved just changing the conductance of this new current *J*_*to*_.

**Figure 6:**
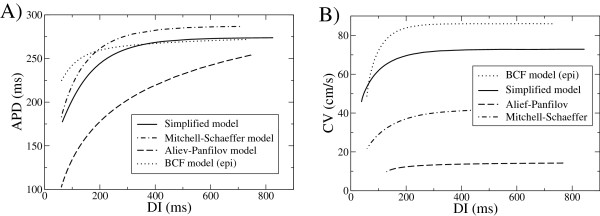
**Comparison of the APD and CV-restitution curves for several simplified models in the literature.** We plot results from our simplified model (solid lines) and the 5 variable BCF model (dotted lines), fitted to epicardial cells, the Mitchell-Schaeffer model (dashed-dotted lines), and the Aliev-Panfilov model (dashed lines).

In Figure 6 we show the APD and CV restitution curves obtained for the different simplified models. For our model and BCF we consider the values fitted to human ventricular epicardial cells, while for the models by Aliev-Panfilov and Mitchell-Schaeffer we consider typical values of the parameters cited in the original papers. The latter do not correspond to human cells.

## Two dimensional simulations

In this subsection, we will check that our model reproduces the complex spiral wave dynamics observed in realistic cardiac models (for a comparison of spiral wave dynamics in several cardiac models, see [37]). In order to do so, we take a piece of uniform epicardium tissue and apply an ectopic activation to study the stability of the artificially created spiral wave and its dynamics. Spiral tips may follow different types of trajectories, from circles to flower like patterns to chaotic meanderings [38,39]. The spiral tip is determined by the algorithm described by Fenton and Karma in the reference [15]. The wave tip is defined as the point where the excitation wavefront meets the repolarization waveback of the AP. This point (*x*_*tip*_,*y*_*tip*_) is the intersection point of the lines *V *=* V*_*iso *_and *∂*_*t*_*V *= 0. The arbitrary choice for *V*_*iso *_only slightly modifies the meander trajectories. In this article, we have chosen *V*_*iso *_as a half of the maximum depolarization potential value, i.e. *V*_*iso *_≈ (*ΔV*)/2.

**Figure 7:**
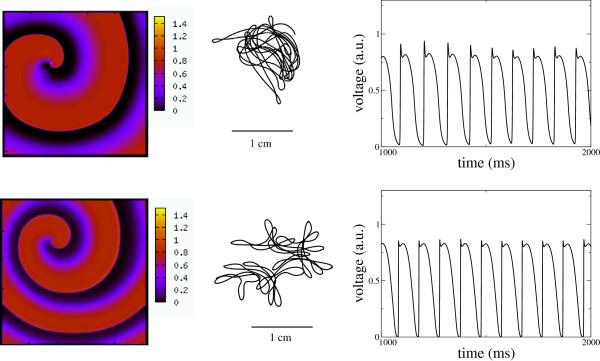
**Comparison of the spiral dynamics obtained with the original LRd model (Upper) and our present model (Lower) with the parameters given in Table **1. On the left it is shown a typical snapshot of the spiral wave; in the middle it is shown the tip trajectory and on the right a time series of the transmembrane voltage taken at a fixed point in the two dimensional domain. Note that the potential for the LRd model has been renormalized to fit in the range [0−1*.*5] in order to ease the comparison. The tissue size is 10x10 cm.

In Figure 7 it is shown the spiral dynamics for the LRd model and our present model. The size of the 2D domain is 10x10 cm. The time integration spans 4,000 ms and only a fraction is shown. The spirals are stable and the spiral tip experiences a meander that is comparable for both the original LRd model and our model. Here we should point out that as the spiral dynamics is not directly related to the APD and CV curves, this comparison constitutes an additional test for the validity of our model. However, given the strong memory effects in the LRd model, a very precise comparison is difficult to make, since the spiral form and dynamics is slowly changing with time. Quantitatively, the period of the spiral wave for the LRd model is *T *= 104*.*8 ms (APD=86.8 ms and DI=18 ms) and for our model fitted for LRd, the spiral period is *T *= 102 ms (APD=83.8 ms and DI=18.2 ms). The high frequency of the spiral waves is the reason for using the four different BCL in the fitting of the AP morphology in section II (especially the high frequency rate at BCL=100 ms). Actually, due to the spiral motion, the period varies slightly from point to point in the two dimensional domain (Doppler shift). The size of the spiral core is also comparable and is of the order of 1cm (see Figure 7).

## Three dimensional simulations and ECG calculations

Cardiac arrhythmias, especially those occurring in the ventricles, are intrinsically three-dimensional phenomena whereas direct experimental observations are only mostly limited to surface recordings. Other techniques like plunge electrodes can and have been used to obtain intramural information, albeit at coarse spatial resolutions [40], and transillumination is another technique that can be also used in some circumstances [41]. Therefore, it is in general difficult to identify the exact nature of the arrhythmic states and the mechanisms causing their initiations. In this respect, computer simulations of the propagating action potential throughout the heart constitute an invaluable tool to get a better insight into these mechanisms.

**Figure 8:**
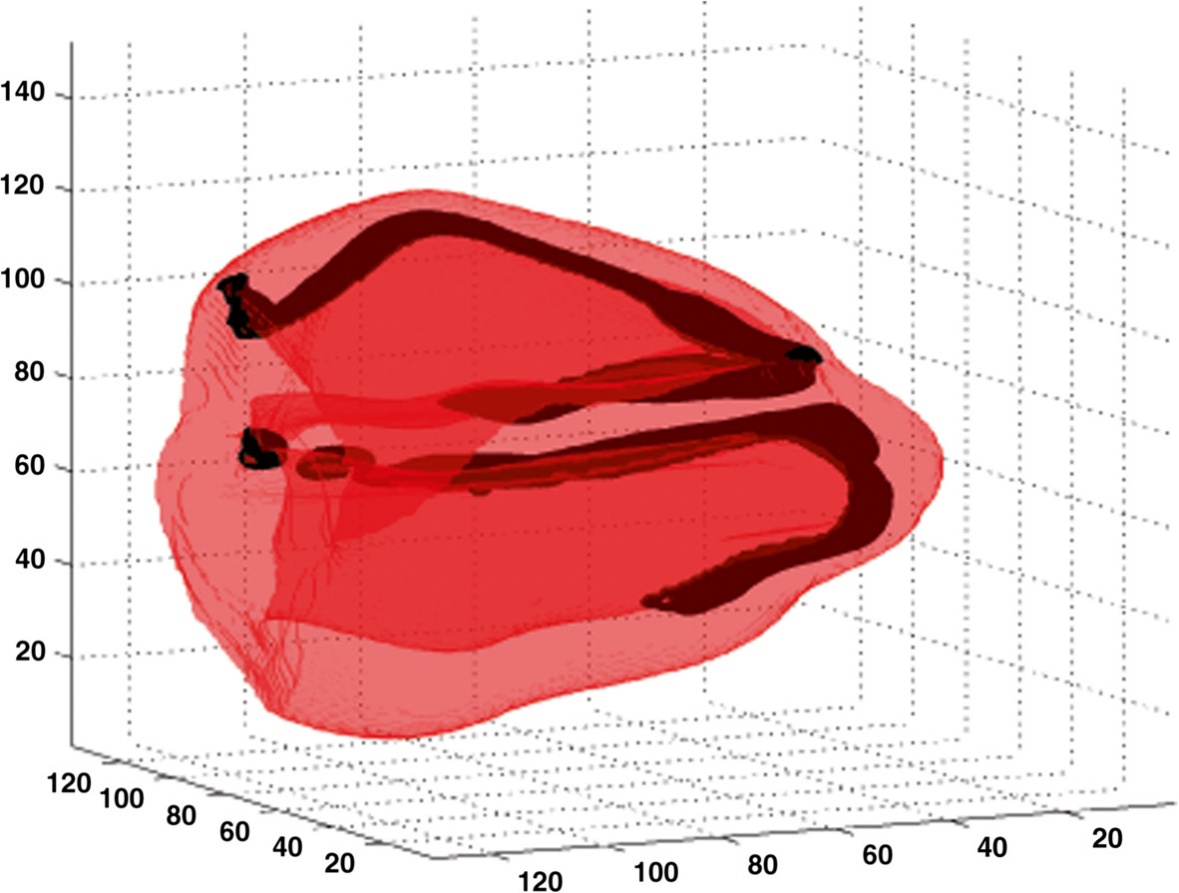
**Geometry of the rabbit heart ventricles.** The dark traces are the locations where the excitation of the ventricles is initiated (time course of 10 ms). Not shown in this figure is the anisotropy of electrical conductivity of the cardiac tissue. The box size is 152x138x130 voxels (dx=dy=dz=0.025cm). The left ventricle corresponds to the lower part of the figure and the right ventricle is located in the upper part of the figure.

In this section we exploit a three dimensional computer model of the rabbit heart ventricles to further compare the original LRd model and our new model. In particular, we compute and compare the pseudo-ECGs [42] resulting from the AP propagation for both models. The details of the numerical scheme used in this section can be found in the paper by Bragard *et al*. [43]. The geometry of the ventricles are corresponds to the rabbit heart [26] and we have also included the anisotropy of the conductivity tensor which is very important in order to achieve realistic simulations of the propagating AP. Here, the numerical grid is composed by cubic voxels of 0*.*025 cm size and the time step of the explicit scheme is set to *dt *= 0*.*01 ms. Using such a small time step ensures to capture the smallest time scale linked to the depolarization of the heart myocytes (≈1 ms). The values for the longitudinal diffusion *D*_∥ _= 10^−3^ cm^2^/ms and transverse diffusion *D*_⊥ _= 6*.*75×10^−5^cm^2^/ms are taken from the paper by Aguel *et al*. [44] and reflect the fact that the conduction velocity is more than three times faster along the fibers than in the plane transverse to the fibers (i.e. transverse isotropic diffusion tensor). In addition to the geometry and the fiber anisotropy, we have modeled the complex fast conduction system (His bundle and Purkinje fibers [45]) by a time dependent external activation along the endocardial layer as it is done in the article by Boulakia *et al*. [46]. Indeed, the time sequence of the depolarization of the ventricular tissue is important for the reconstruction of the ECG. Here, in the rabbit heart ventricles, we have represented in black the location where the activation is initiated (see Figure 8). The detail of the sequence of the firing is as follows: At t=0, the fibers between the base and the mid-septum are fired; at t=5 ms, the fibers between the mid-septum and the apex are fired and, finally, the remaining fibers are fired at t=10 ms. In the present paper, the ECG has been reconstructed using the heart dipole technique [47]. This technique consists in solving the forward problem of electro-cardiography with a monodomain approximation [46] and assuming that the torso is an homogenous medium. This is the simplest manner to compute the pseudo-ecgs but still gives satisfactorily qualitative results. The method consists in adding all the microscopic dipoles (created at the depolarization fronts) into a single vector which is called the heart dipole vector as follows:

(11) $$\vec{J}=\int_{V}\vec{r}\;\nabla \cdot (D\nabla V)\;dV\;,$$

where in Equation (11), V represents the integration volume (ventricles) and $\vec{r}$ is the vector joining the geometrical center of the heart to the microscopic dipoles created by the moving fronts. The next step consists in projecting the heart vector on some standard directions in order to compute the different derivations of the ECG. In our case, because of the crude approximation used for the torso, we are unable to compute the chest leads [46]. As the main interest of this section was to compare our model with the LRd model, we will only compute the standard lead I and check if they are qualitatively the same.

**Figure 9:**
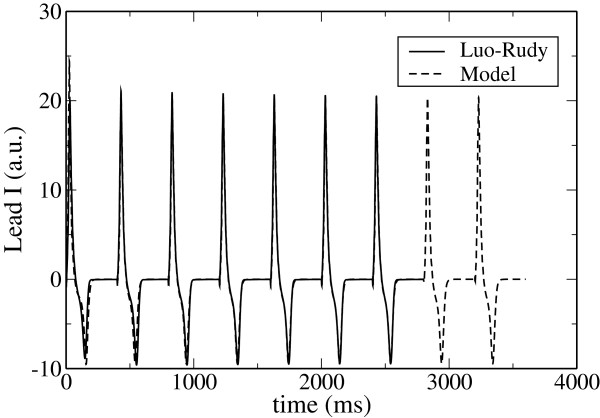
**Comparison of the Lead I of the pseudo-ECG computed from the 3D simulations.** We have compared the cases of the original LRd model (solid line) and our simplified model (dashed line).

For the comparison of the pseudo-ecgs we have stimulated both our model and the LRd model at a period of BCL=400 ms during several beats. In the case of the LRd model, and because this model is known to have memory effects associated to the slow dynamics of ion concentrations, we have started the simulation from an initial condition obtained from a separate one dimensional simulation of one thousand beats. The electrical organized activity associated with the AP propagation is well captured in the corresponding pseudo-ECGs as shown in Figure 9. We observe that after few beats both models (ours and LRd) converge as expected. It should be noticed that the T-wave indicating the repolarization phase of the ventricles is inverted in both ECGs in Figure 9. This is due to the fact that our description of the ventricles lacks to include the transmural heterogeneities, i.e. different APDs of the cardiac myocytes in the epi-, mid- and endocardium tissues [46,48]. From the computational speed point of view, the difference between our model and the LRd model is striking. In order to have a fair comparison, we have simulated both models on the same single processor, thus avoiding differences linked to parallelization speed variations. For the LRd model the simulation lasted for 68 hours, while only 4h30’ for our model. Therefore a gain of approximately a factor twenty in computational speed. As mentioned in [46], if we want to address the inverse problem of electro-cardiography the speed is a crucial factor to take into account because one has to solve many times the forward problem to get an approximation of the inverse problem. The use of a simplified model is one way to deal with the speed issue.

## Conclusions and further works

In this paper we have analyzed a semi-physiological cardiac model that has been used previously to study reexcitation in a medium with dispersion of repolarization [25]. The model reproduces well the action potential morphology of different cardiac cells from experimental data and also from more complete physiological models.

The CV-restitution and the pseudo-ECG’s in a realistic model of heart ventricles have also been satisfactorily compared. One concludes that to study AP propagation our present simplified model is a good alternative to the most costly physiological models. The gain in computer speed is around a factor of twenty and the need for computer memory (RAM) is also greatly reduced. In certain situations it also allows to obtain analytically some characteristics of propagation [25], which, in a realistic cardiac model, would be intractable. A limitation of the model is the absence of memory due to the lack of the slow dynamics of ion concentrations. This makes unfeasible to fit simultaneously dynamic and S1-S2 restitution curves and may not give a good comparison with realistic models under situations where the stimulation frequency is abruptly changed. In the near future, we plan to refine the present three dimensional simulation to include the important transmural heterogeneities [49], in order to study transmural reexcitations and to obtain more realistic ECGs from the 3D model.

## Competing interests

The authors declare that they have no competing interests.

## Authors’ contributions

All authors read and approved the final manuscript.

## References

[B1] Hodgkin A Huxley A A quantitative description of membrane current and its application to conduction and excitation in nerve J Physiol 1952 117 500 544 12991237 10.1113/jphysiol.1952.sp004764 PMC1392413

[B2] Fenton FH Cherry EM Models of cardiac cell Scholarpedia 2008 3 1868 10.4249/scholarpedia.1868

[B3] Puglisi JL Bers DM LabHEART: an interactive computer model of rabbit ventricular myocytes ion channels and Ca transport Am J Physiol 2001 281 C2049 C2060 10.1152/ajpcell.2001.281.6.C2049 11698264

[B4] Luo C Rudy Y A dynamic model of the cardiac ventricular action potential - Simulations of ionic currents and concentration changes Circ Res 1994 74 1071 1097 10.1161/01.RES.74.6.1071 7514509

[B5] Pandit SV Clark RB Giles WR Demir SS A mathematical model of action potential heterogeneity in adult rat left ventricular myocytes Biophys J 2001 81 3029 3051 10.1016/S0006-3495(01)75943-7 11720973 PMC1301767

[B6] Winslow RL Rice J Jafri S Marban E O’Rourke B Mechanisms of altered excitation-contraction coupling in canine tachycardia-induced heart failure. II. Model studies Circ Res 1999 84 571 586 10.1161/01.RES.84.5.571 10082479

[B7] Priebe L Beuckelmann DJ Simulation study of cellular electric properties in heart failure Circ Res 1998 82 1206 1223 10.1161/01.RES.82.11.1206 9633920

[B8] ten Tusscher K Noble D Noble PJ Panfilov AV A model for human ventricular tissue Am J Physiol Heart Circ Physiol 2004 286 H1573—H1589 14656705 10.1152/ajpheart.00794.2003

[B9] Iyer V Mazhari R Winslow RL A computational model of the human left-ventricular epicardial myocyte Biophys J 2004 87 1507 1525 10.1529/biophysj.104.043299 15345532 PMC1304558

[B10] Grandi E Pasqualini FS Bers DM A novel computational model of the human ventricular action potential and Ca transient J Mol Cell Cardiol 2010 48 112 121 10.1016/j.yjmcc.2009.09.019 19835882 PMC2813400

[B11] Clancy CE Rudy Y Na+ channel mutation that causes both Brugada and long-QT syndrome phenotypes: a simulation study of mechanism Circulation 2002 105 1208 1213 10.1161/hc1002.105183 11889015 PMC1997279

[B12] FitzHugh R Impulses and physiological states in theoretical models of nerve membrane Biophysical J 1961 1 445 466 10.1016/S0006-3495(61)86902-6 PMC1366333 19431309

[B13] Nagumo J Arimoto S Yoshizawa S An active pulse transmission line simulating nerve axon Proc IRE 1962 50 2061 2070

[B14] Aliev RR Panfilov AV A simple two-variable model of cardiac excitation Chaos: Solitons Fractals 1996 7 293 10.1016/0960-0779(95)00089-5

[B15] Fenton F Karma A Vortex dynamics in three dimensional continuous myocardium with fiber rotation: Filament instability and fibrillation Chaos 1998 8 20 47 10.1063/1.166311 12779708

[B16] Mitchell CC Schaeffer DG A two-current model for the dynamics of cardiac membrane Bull Math Biol 2003 65 767 793 10.1016/S0092-8240(03)00041-7 12909250

[B17] Cherry EM Fenton FH Suppression of alternans and conduction blocks despite steep APD restitution: electrotonic, memory, and conduction velocity restitution effects Am J Physiol Heart Circ Physiol 2004 286 H2332—H2341 14751863 10.1152/ajpheart.00747.2003

[B18] Cherry EM Ehrlich JR Nattel S Fenton FH Pulmonary vein reentry–properties and size matter: insights from a computational analysis Heart Rhythm 2007 4 1553 1562 10.1016/j.hrthm.2007.08.017 18068635

[B19] Bueno-Orovio A Cherry EM Fenton FH Minimal model for human ventricular action potential in tissue J Theor Biol 2008 253 544 560 10.1016/j.jtbi.2008.03.029 18495166

[B20] Cytrynbaum E Keener JP Stability conditions for traveling pulse: modifying the restitution hypothesis Chaos 2002 12 788 799 10.1063/1.1503941 12779607

[B21] Antzelevitch C Role of spatial dispersion of repolarization in inherited and acquired sudden cardiac death syndromes Am J Physiol Heart Circ Physiol 2007 293 H2024 H2038 10.1152/ajpheart.00355.2007 17586620 PMC2085107

[B22] Brugada P Brugada J Role of spatial dispersion of repolarization in inherited and acquired sudden cardiac death syndromes J Am Coll Cardiol 1992 20 1391 1396 10.1016/0735-1097(92)90253-J 1309182

[B23] Jervell A Lange-Nielsen F Congenital deaf-mutism, functional heart disease with prolongation of the Q-T interval and sudden death A Heart J 1957 54 59 68 10.1016/0002-8703(57)90079-0 13435203

[B24] Li GR Feng J Yue L Carrier M Transmural heterogeneity of action potentials and Ito1 in myocytes isolated from the human right ventricle Am J Physiol 1998 275 H369 H377 9683422 10.1152/ajpheart.1998.275.2.H369

[B25] Cantalapiedra IR Peñaranda A Echebarria B Bragard J Phase-2 reentry in cardiac tissue: role of the slow calcium pulse Phys Rev E 2010 82 011907 10.1103/PhysRevE.82.011907 20866648

[B26] Vetter FJ McCullogh AD Three-dimensionnal analysis of regional cardiac function: a model of the rabbit ventricular anatomy Prog Biophys Mol Biol 1998 69 157 183 10.1016/S0079-6107(98)00006-6 9785937

[B27] Nabauer M Beuckelmann DJ Uberfuhr P Steinbeck G Regional differences in current density and rate-dependent properties of the transient ventricular electrophysiology Am J Physiol 1996 292 H43—H55 10.1161/01.cir.93.1.168 8616924

[B28] Cantalapiedra IR Peñaranda A Mont L Brugada J Echebarria B Reexcitation mechanisms in epicardial tissue: role of I(to) density heterogeneities and I(Na) inactivation kinetics J Theor Biol 2009 259 850 859 10.1016/j.jtbi.2009.04.021 19410581

[B29] Peñaranda A Cantalapiedra IR Echebarria B Slow pulse due to calcium current induces phase-2 reentry in heterogeneous tissue Comput Cardiol 2010 37 661 664

[B30] Faber GM Rudy Y Action potential and contractility changes in Na+ overloaded cardiac myocytes: a simulation study Biophys J 2000 78 2392 2404 10.1016/S0006-3495(00)76783-X 10777735 PMC1300828

[B31] Webpage with the codes used for the fit and further information http://www-fa.upc.es/websfa/eupb/NOLIN/CARDIAC/Simp_model.html

[B32] Drouin E Charpentier F Gauthier C Laurent K LeMarec H Electro physiologic characteristics of cells spanning the left ventricular wall of human heart: evidence for presence of M cells J Am Coll Cardiol 1995 26 185 192 10.1016/0735-1097(95)00167-X 7797750

[B33] Morgan JM Cunningham D Rowland E Dispersion of monophasic action potential duration: demonstrable in humans after premature ventricular extrastimulation but not in steady state J Am Coll Cardiol 1992 19 1244 1253 10.1016/0735-1097(92)90331-G 1373420

[B34] Yue AM Franz MR Roberts PR Morgan JM Global endocardial electrical restitution in human right and left ventricles determined by noncontact mapping J Am Coll Cardiol 46 1067 1075 16168293 10.1016/j.jacc.2005.05.074

[B35] LRd ventricular cell model (guinea-pig-type), source code [ http://rudylab.wustl.edu/research/cell/methodology/cellmodels/LRd/code.htm]

[B36] Bernus O Wilders R Zemlin CW Verschelde H Panfilov AV A computationally efficient electrophysiological model of human ventricular cells Am J Physiol Heart Circ Physiol 2002 282 H2296—2308 12003840 10.1152/ajpheart.00731.2001

[B37] Shajahan TK Nayak AR Pandit R Spiral-wave turbulence and its control in the presence of inhomogeneities in four mathematical models of cardiac tissue PLoS ONE 2009 4 e4738 10.1371/journal.pone.0004738 19270753 PMC2650787

[B38] Davidenko JM Pertsov AM Salomonsz R Baxter WT Jalife J Stationary and drifting spiral waves of excitation in isolated cardiac muscle Nature 1992 355 349 351 10.1038/355349a0 1731248

[B39] Fenton FH Cherry EM Hastings HM Evans SJ Multiple mechanisms of spiral wave breakup in a model of cardiac electrical activity Chaos 2002 12 3 852 892 10.1063/1.1504242 12779613

[B40] Allison JS Qin H Dosdall DJ Huang J Newton JC Allred JD Smith WM Ideker RE The transmural activation sequence in porcine and canine left ventricle is markedly different during long-duration ventricular fibrillation J Cardiovasc Electrophysiol 2007 18 306 312 10.1111/j.1540-8167.2007.00963.x 17916154

[B41] Baxter WT Mironov SF Zaitsev AV Jalife J Pertsov AM excitation waves inside cardiac muscle using transillumination Biophys J 2001 80 516 530 10.1016/S0006-3495(01)76034-1 11159422 PMC1301253

[B42] Bernabeu MO Corrias A Pitt-Francis J Rodriguez B Bethwaite B Enticott C Garic S Peachey T Tan J Abramson D Gavaghan D Grid computing simulations of ion channel block effects on the ECG using 3D anatomically-based models Comput Cardiol 2009 36 213 216

[B43] Bragard J Marin S Cherry E Fenton F Validation of a model of cardiac defibrillation To appear in Springer-book (2012)

[B44] Aguel F Eason J Trayanova N Advances in modeling cardiac defibrillation Int J Bifurcation Chaos 2003 13 3791 3805 10.1142/S0218127403008892

[B45] Katz AM Physiology of the heart 2005 Philadelphia: Lippincott Williams & Wilkins

[B46] Ambrosi D, Quarteroni A, Rozza G Numerical simulations of electrocardiograms 2011 Springer

[B47] Constanzo LS Physiology 2002 Philadelphia, P A: W.B. Saunders

[B48] Gussak I Antzelevitch C Cardiac Repolarization: Bridging Basic and Clin Sci 2003 New York: Humana Press, Springer

[B49] Abd Allaha ESH Aslanidic OV Telleza JO Yannia J Billeterd R Zhangc H Dobrzynskia H Boyett MR Postnatal development of transmural gradients in expression of ion channels and Ca2+ handling proteins in the ventricle J Mol Cell Cardiol 2012 53 2 145 155 10.1016/j.yjmcc.2012.04.004 22537893

